# Immediate postoperative complications after lateral ridge augmentation – a clinical comparison between bone shell technique and sticky bone

**DOI:** 10.25122/jml-2021-0347

**Published:** 2022-04

**Authors:** Stefania Andrada Iancu, Daniel Referendaru, Ilinca-Antigona Iancu, Anamaria Bechir, Horia Mihail Barbu

**Affiliations:** 1. European Centre of Oral Implantology, Bucharest, Romania; 2. Department of Prosthodontics, Faculty of Dental Medicine, Titu Maiorescu University, Bucharest, Romania; 3. Discipline of Physiology, Carol Davila University of Medicine and Pharmacy, Bucharest, Romania; 4. Department of Dental Specialties, Titu Maiorescu University, Bucharest, Romania; 5. Oral Implantology Department, Faculty of Dental Medicine, Titu Maiorescu University, Bucharest, Romania

**Keywords:** bone block, bone shell technique, sticky bone, lateral ridge augmentation, surgical morbidity, trismus, hematoma, neurosensory disturbance

## Abstract

Nowadays, implant dentistry is strongly interconnected to bone augmentation procedures. Lateral ridge augmentation is often an imperative treatment stage for successful, prosthetic-driven implant placement. This study aimed to comparatively analyze the immediate postoperative complications of two horizontal bone grafting procedures: sticky bone and bone shell technique. Records of patients with lateral ridge augmentation were analyzed to identify immediate postoperative complications. The study group included 80 patients divided into 40 control (bone-shell technique – BS) and 40 tests (sticky bone –SB). More patients reported moderate and severe pain in the BS – group (11 patients – 27.5%) than in the SB group (6 patients – 15%). In the BS group, the incidence of severe and moderate trismus, neurosensory disturbances, and important hematoma was higher. There was an increased inflammatory response following the bone shell technique, while the sticky bone technique proved reduced surgical morbidity. There was no difference between the two groups in the risk of dehiscence or infection.

## Introduction

Bone reconstruction for implant placement varies in numerous techniques and materials, each of them bringing advantages and specific qualities for different types of bone defects [1, 2]. Research is meant to enhance different biomaterials and surgical procedures for a long-term successful outcome of the implants and the prosthetic restorations [2, 3]. In terms of osteogenesis, all techniques known so far promise new bone formation, independent of the complexity of the clinical case. Improvements in different surgical protocols aim to shorten the surgery duration, reduce the number of treatment stages, the risk of intra- and postoperative complications, and the overall morbidity of the procedure [3–5]. Surgeons have to weigh the advantages of different surgical methods and biomaterials, the degree of tissue injury, the benefits and risks of minimally invasive techniques to obtain successful outcomes.

Reduced postoperative complications mean a reduced stress response to surgery and accelerated recovery. Minimum postoperative morbidity brings physical and psychological comfort to the patients, which increases trust in dental treatment. However, surgical morbidity should not compromise the decision of adequate bone grafting protocol. It should be the secondary rationale for techniques that are equally mastered by the surgeon and provide the same results. An incorrect minimally invasive protocol (reduced mucoperiosteal flap design, tunnel technique performed by inexperienced practitioners) could increase surgical morbidity and lead to graft failure [4, 5].

This study aimed to comparatively evaluate the incidence of immediate postoperative complications after two different bone augmentation procedures (BS – bone shell technique *vs.* SB – sticky bone).

## Material and Methods

### Study design and population

The research was structured as a retrospective cohort study of 80 surgeries of horizontal ridge augmentation, following the STROBE (Strengthening the Reporting of Observational Studies in Epidemiology) guidelines [6]. The postoperative symptomatology and signs of two different horizontal bone grafting procedures were comparatively analyzed. The analysis was performed using postoperative pictures of the patients with lateral edentulous ridge augmentation and their medical files, which included objective and subjective findings. The immediate postoperative complications were registered in analog and digital form in the patient documentation. The entire database of patients and radiological examinations belonged to Prof. Dr. Barbu Dental Clinic, located in Bucharest, Romania.

### Study population

Eligible subjects for this study were chosen upon a detailed analysis of the medical records of patients who underwent lateral ridge augmentation with sticky bone (SB) and bone-shell technique (BS).

Inclusion criteria:

• Lateral ridge augmentation performed with BS or SB;
• Bone augmentation limited to a single quadrant (covering an edentulous area of 1 to 3 missing teeth);
• Patients with stable mental health, no previous records of anxiety/depression;
• Patients with multiple staged surgeries and/or simultaneous bone grafting and implant placement.

Exclusion criteria:

• Patients with a recent or anterior diagnosis of depression, generalized anxiety, obsessive-compulsive disorder, insomnia;
• Medication or health conditions that contraindicated surgical procedures (bisphosphonates therapy, radiotherapy in region of the head and neck, uncontrolled diabetes);
• Complex, one-staged full-mouth rehabilitation surgeries;
• Patients who postponed the appointments in the first week after the surgery;
• Absence of informed consent for participation in this study.

### Surgical protocol

All surgeries began with local anesthesia, performed with articaine hydrochloride with adrenaline 1:100000 (Ubistesin Forte, 3M ESPE, Seefeld, Germany). For the maxilla PSA (posterior superior alveolar), nerve block with additional palatal nerve block was performed, while for the mandible, Weisbrem's technique (inferior alveolar nerve, together with the lingual and buccal nerve block) was implemented. For both techniques, the mucoperiosteal flap elevation followed multiple cortical plate perforations to increase the angiogenesis potential of the recipient bed for the future bone graft.

The decision to simultaneously insert the implant depended on the width of the residual edentulous ridge. If primary implant stability could be achieved, the implant osteotomy was performed, and further bone graft would build missing bone volume. In the absence of adequate primary implant stability, the horizontal augmentation procedures were the prime concern, and the implant insertion followed months later.

For the bone shell technique, a cortico-cancellous bone block was harvested from the external oblique ridge with the aid of the micro-saw-shaped tip OT7S and the ultrasonic equipment (Piezosurgery, Mectron, Carasco, Italy). The alternative harvesting method was a straight handpiece and a diamond disk (Frios MicroSaw, Dentsply Sirona, Charlotte, NC, USA). The surgeon used only thin bone segments for the bone shell technique, which were obtained after the initial thicker cortico-cancellous bone block was longitudinally split into two. Depending on the size of the recipient bed to be augmented, both thinner bone blocks were used, or just a single bone piece served for bone grafting.

To avoid flap perforations during the healing period, the entire sharp contour of the bone block was smoothed with a round diamond bur. Then, rigid fixation of the bone block to the buccal plate of the edentulous ridge was performed with the aid of 5 mm, 7 mm, or 9 mm long osteosynthesis screws (Devemed GmbH, Tuttlingen, Germany). Specific to this bone grafting method, a 3–4 mm empty space was intentionally left between the buccal plate and the bone block. Autogenous bone chips were slightly compressed inside this bone frame until the entire space was filled with autogenous bone particles.

For maxillary ridge augmentation, the bone blocks were harvested from the anterior wall of the maxillary sinus for cases when sinus floor elevation was simultaneously performed with lateral crest augmentation. For the other cases which required only horizontal bone grafting, the zygomatic buttress served as the donor site.

The augmentation procedures performed with sticky bone were particular through the bone aggregate incorporated in autologous plasma. The aggregate included exclusive autogenous bone chips or a mixture of autologous bone (75%) with particulate bovine bone (25%). The harvesting method for autologous bone chips varied from the bone scraper, ACM drills (Auto Chips Maker, Neo-Biotech, Seoul, South Korea), rongeur forceps (especially for donor sites located in the maxilla), or the implant osteotomy drills at low speed.

**Figure 1.**
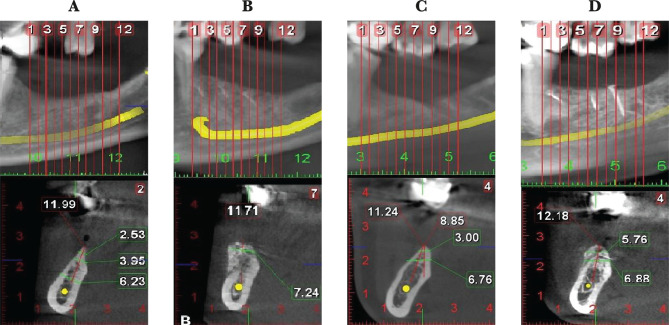
CBCT sections of the initial (A, C) and the augmented surgical site, 6 months after the surgery, with sticky bone (B) and bone block (D).

All subjects were prescribed the same postoperative medication. Steroidal anti-inflammatory drugs (dexamethasone sodium phosphate 8 mg) were prescribed 3 days (the first day prior to surgery, the second, and the third day after the augmentation procedure), additional non-steroidal anti-inflammatory drugs (dexketoprofen 25 mg) were given when needed. The same antibiotics (amoxicillin and clavulanic acid 1 g every 12 h) were prescribed for 7 days to all patients. The postoperative evolution was assessed at appointments at 3,7, 10, and 21 days after the surgery. Radiological evaluation of the grafted area was made on CBCT performed 6 months after the surgery. A comparison of the initial and postoperative surgical site is represented in Figure 1 A–D.

### Clinical postoperative examination – subjective and objective findings of inherent postoperative complications

During recall appointments, a clinical examination was performed, and the following immediate postoperative complications were evaluated:

1. Pain;
2. Trismus;
3. Hematoma;
4. Neurosensory disturbance;
5. Postoperative dehiscence and infection;
6. Postoperative hemorrhage.

A single operator registered the postoperative clinical findings in the patients' medical files.

Pain level was evaluated using a questionnaire with a visual analog scale (VAS) ranging from 0 to 10. When describing the pain level, the patients had the following options: "none", "mild", "moderate", and "severe". The pain level perceived by the patients in the first five days after the surgery was significant to the study.

Postoperative trismus was evaluated by measuring the maximal opening of the mouth on the third day following surgery. An interincisal measurement between 10 mm and 25 mm was the reference for moderate trismus, and an interincisal opening <10 mm for severe trismus.

Only important hematomas, which required drainage, were considered in this study. The patients reported tenderness and discomfort, and the intraoral examination revealed an increased tension on the sutures and flap edges. Surgical drainage of the hematoma was performed on the first day after the surgery, inserting a spatula along the vertical release incision of the mucoperiosteal flap. At the same time, slight pressure on the soft tissue covering the hematoma was applied.

The neurosensory disturbance was usually described as hypoesthesia and paresthesia, localized in the mental region. A complex of γ-linolenic acid, alpha-lipoic acid, selenium, vitamin B-complex and vitamin E (Alanerv, Alfasigma, Bologna, Italy) was prescribed (1 capsule daily) for 3 weeks or more if needed.

Regarding the bleeding complications, the postoperative hemorrhage was treated by additional sutures performed in the first hours after the surgery. Wound dehiscence was treated differently according to graft exposure alone or with symptoms and signs of infection. For the exposure of a reduced area of autologous bone graft material, the operator chose expectant management, while infected augmentation sites were prone to curettage with the removal of the infected tissue and bone graft material. 

## Results

The present study included 80 surgeries (40 test – sticky bone and 40 control – bone shell technique) of horizontal ridge augmentation performed by the same surgeon (H.B.) between 2011 and 2019. The gender distribution showed no important difference between the two groups (test group – 28 female and 12 male patients and control group – 29 female and 11 male patients) with chi-square p=0.8. The entire group of patients had a mean age of 58.3±13.4 years. The mean age of the test group was 51.0±11.9 years and 47.4±9.7 years in the control group. Age distribution was performed according to the Shapiro-Wilk test, following a parametric test (two-tailed t-test). The age distribution between the two samples showed no statistical significance (p-value 0.14). A total of 32 smoker patients underwent horizontal ridge augmentation surgeries. The test group included 17 smokers and 23 non-smoker patients, and the control group included 15 smokers and 25 non-smoker patients. There was no statistical significance regarding the smoking habit of the patients between the two groups (p=0,65).

### Postoperative pain

Within the bone blocks (BS) group, 10 subjects described a painless healing period (25%) immediate after the surgical procedure, 19 subjects (47%) related mild pain, 9 patients described moderate pain (23%), and 2 patients severe pain (5%). In the sticky bone (SB) group, 18 patients had no pain (45%), 16 patients had mild pain (40%), 5 subjects reported moderate pain (13%), and one patient described severe pain (2%) (Table 1).

**Table 1.** Pain level assessment between the control (BS) and the test (SB) group.

**Pain level scale/type of augmentation**	**None (0)**	**Mild pain (1–3)**	**Moderate pain (4–6)**	**Severe pain (7–10)**
**Bone block**	10 (25%)	19 (47%)	9 (23%)	2 (5%)
**Sticky bone**	18 (45%)	16 (40%)	5 (13%)	1 (2%)

The number of patients with moderate and severe pain was higher in the bone block group (11 patients – 27.5%) than in the sticky bone group (6 patients – 15%). Another element taken into consideration in each study group was the harvesting method. Thus, in the bone block group, all 10 patients with no pain (25%) were treated with piezosurgery equipment. Moderate pain levels were reported in 8 cases with the diamond disk harvesting method (20%) and 1 case (2.5%) with an ultrasonic bone surgery device. The 2 cases with severe pain belonged to the subclass with the diamond disk harvesting method (Table 2).

**Table 2.** Pain level associated with bone graft harvesting method in the control group (BS-technique).

**Pain level/ harvesting method**	**Diamond disk (18 cases)**	**Piezosurgery (22 cases)**
**None**	0	10
**Mild pain**	8	11
**Moderate pain**	8	1
**Severe pain**	2	0

In the test group (SB technique), the only cases with moderate pain (5 patients – 12.5%) and severe pain (1 patient – 2.5%) were associated with the harvesting method using the implant osteotomy drills when implant placement was performed simultaneously with bone augmentation (Table 3).

**Table 3.** Pain level correlated with the autologous bone chips harvesting method in the test group (SB-technique).

**Pain level/ harvesting method**	**ACM drills (22 cases)**	**Bone scraper (6 cases)**	**Implant osteotomy drills (6 cases)**	**Rongeur forceps (6 cases)**
**None**	12	3	0	3
**Mild pain**	10	3	0	3
**Moderate pain**	0	0	5	0
**Severe pain**	0	0	1	0

### Trismus

After the bone shell technique, 26 patients (65%) experienced moderate trismus, and 2 patients presented severe trismus (5%). In the sticky bone group, 22 patients (55%) presented moderate trismus, and only 1 patient had severe trismus (2.5%) (Table 4).

**Table 4.** Incidence of postoperative trismus between the test (SB) and control (BS) group.

**Trismus**	**BS**	**SB**
**Normal mouth opening**	12 (30%)	17 (42.5%)
**Moderate trismus (mouth opening 10–25 mm)**	26 (65%)	22 (55%)
**Severe trismus (mouth opening <10 mm)**	2 (5%)	1 (2.5%)

When correlating the incidence of trismus with the surgical site, in both study groups, the incidence was higher for bone grafting procedures performed in the mandible (87% for BS group and 83% for SB group) than in the maxilla (Table 5).

**Table 5.** Topographic correlation of the incidence of postoperative trismus in control and test groups.

**Surgical site (donor and recipient)**	**BS (control group)**	**SB (test group)**
**Mandible**	26 (87%)	19 (83%)
**Maxilla**	4 (13%)	4 (17%)

### Hematoma

Important hematomas which needed drainage were present in 9 cases (22.5%) in the BS group and 7 cases (17.5%) in the SB group.

### Neurosensory disturbance

From the entire study group (n=80), 8 patients (10%) presented temporary neurosensory disturbance, with a minimum of 7 days to a maximum of 5 weeks. In all cases, complete healing occurred, and the patients achieved normal tactile sensation of the lower lip. 

In the BS group, 6 patients (15%) presented neurosensory disturbance, while in the SB group, only 2 patients (5%) presented neurosensory disturbance. In all cases, the affected area was localized in the mandible, in the mental region.

### Dehiscence and infection

Dehiscence alone or associated with graft infection occurred in 10 cases (12.5%) out of 80 bone grafting procedures. In the BS group, there were 3 cases of surgical dehiscence without infection and 2 cases of dehiscence with signs and symptoms of infection (n=5, all in the mandible). In the SB group, a number of 3 flap dehiscence and 2 bone graft infections were registered (n=5, two infections and one flap dehiscence in the maxilla and two flaps dehiscence in the mandible).

### Postoperative hemorrhage

Postoperative bleeding occurred in 2 cases (5%), one with sticky bone and the other one with bone block augmentation. In both cases, the surgeries were performed in the maxilla, with minor bleeding complications and required suture along the vertical release incision within the first hours after the procedure.

## Discussion

The surgical morbidity of an augmentation procedure can be the rationale for electing a specific treatment option. Nowadays, implant dentistry is concentrated on high esthetic and functional results with minimum discomfort in the shortest time possible. For an efficient treatment, edentulous ridges reduced in volume may be treated with angulated implants to avoid additional bone augmentation procedures, providing at the same time the function of the dento-maxillary system [7, 8]. However, the ideal prosthetic position of the implant in the edentulous site is often impossible without bone augmentation procedures [2, 9, 10].

Important atrophies of the edentulous ridges sometimes restrain simple treatment possibilities, imposing different types of vertical and horizontal bone grafting procedures [11–13]. Studies regarding horizontal ridge augmentation with sticky bone or bone shell technique (F. Khoury) proved minor differences in the bone width gained 6 months after the procedure [14]. Thus, the surgical morbidity with all the consequences perceived by the patient during the healing period may be a criterion for choosing one procedure instead of the other.

A study performed on 27 lateral ridge augmentation surgeries compared pain levels after guided bone regeneration (GBR) with particulate bovine bone and bone block grafting. The patients received steroidal and non-steroidal anti-inflammatory drugs similar to our study. The results showed no statistical significance between the two groups concerning postsurgical pain level, with generally low postoperative pain perception [15, 16]. Similarly, most of the patients from our cohort described having no pain at all or just mild pain in the first days. A higher number with low pain perception (none or mild) was recorded in the test group (SB) than in the control group (BS). A detailed analysis of the harvesting method for autologous bone showed a difference between diamond disks and the micro-saw OT7S with the piezosurgery unit, promoting the advantages of ultrasonic devices described in many studies [17–19]. In the test group (SB), 6 patients described higher pain levels after bone harvesting with the implant osteotomy drills, which may be related to the additional surgical wound due to simultaneous implant placement.

Regarding the neurosensory disturbance, other studies mention an incidence of 7% [20] to 8% [21] of temporary hypoesthesia of the mental nerve for bone grafting procedures with both donor and recipient sites in the mandible. In our cohort, there was an incidence of 10% (n=8/n=80). There was a difference between control and test, with a higher incidence of temporary neural dysfunction in the bone shell group than sticky bone. A similarity to the study performed by Sakkas on 155 surgeries is the temporary nature of the lesion [20]. None of our patients had permanent damage to any trigeminal nerve. The postoperative neurosensory disturbance is often linked to the harvesting process rather than bone grafting [22].

Postoperative trismus was more frequent after surgeries with bone-shell technique than sticky bone. An important correlation in both groups was the surgical site, with a higher incidence of bone augmentation procedures performed in the mandible. The inferior alveolar nerve block and prolonged surgical procedures are the main etiologic factors of the restricted mouth opening [21, 23–25].

There was a reduced incidence of postoperative hemorrhage, exclusive to maxillary augmentations, in the test group. The main etiological factors were the absorption of vasoconstrictor included in the local anesthetic, as well as a possible neglect of postoperative recommendations (forbidden strong mouth rinsing).

Hematoma drainage was performed to reduce the discomfort and the risk of infection. Important hematomas were found in a higher number in the control group (BS) than test (SB), and these were not correlated to other types of complications (dehiscence and infection).

Flap dehiscence occurred in equal numbers in both groups (n=5 in the test and n=5 in control). In some cases, the surgical wound dehiscence was temporary, and further postoperative evolution within the next weeks revealed normal healing of the surgical site. The infectious complications began with bone graft exposure and directly correspond to the cases with severe postoperative pain levels.

Some limitations of the study are the retrospective nature of the research and the characteristics of the data collected. Both subjective and objective findings are registered with possibly confounding factors that cannot be appropriately controlled (*e.g.*, the operator influence). Thus, the results of this research should be interpreted with caution.

## Conclusion

 Within the limitations of this study, SB and BS proved similar outcomes regarding the immediate postoperative complications, with minor differences in favor of the SB technique. In the BS group, patients experienced a slightly higher surgical morbidity, which was perceived in the first days after the surgery.

## Acknowledgments

### Conflict of interest

The authors declare no conflict of interest.

### Ethical approval

This study was approved by the Institutional Review Board of Titu Maiorescu University, Bucharest, Romania (approval ID: UTM03FEB20-MD19).

### Consent to participate

Written informed consent was obtained from the participants.

### Authorship

SAI contributed to conceptualizing the study and data curation. SAI and IAI contributed to the methodology, writing, and original draft preparation. DR and SAI contributed to the software. HMB and DR contributed to validation, writing, review, and editing. IAI contributed to formal analysis. SAI and HMB contributed to the investigation. HMB contributed with resources and supervision.

All authors have read and agreed to the published version of the manuscript.

## References

[1] Moussa NT, Dym H (2020). Maxillofacial Bone Grafting Materials.. Dent Clin North Am..

[2] Chiapasco M, Casentini P (2018). Horizontal bone-augmentation procedures in implant dentistry: prosthetically guided regeneration.. Periodontology 2000..

[3] Zhao R, Yang R, Cooper PR, Khurshid Z (2021). Bone Grafts and Substitutes in Dentistry: A Review of Current Trends and Developments.. Molecules..

[4] Kim HS, Kim YK, Yun PY (2016). Minimal invasive horizontal ridge augmentation using subperiosteal tunneling technique.. Maxillofac Plast Reconstr Surg..

[5] Deeb GR, Wilson GH, Carrico CK, Zafar U (2016). Is the Tunnel Technique More Effective Than Open Augmentation With a Titanium-Reinforced Polytetrafluoroethylene Membrane for Horizontal Ridge Augmentation?. Journal of Oral and Maxillofacial Surgery..

[6] Von Elm E, Altman DG, Egger M, Pocock SJ (2007). The Strengthening the Reporting of Observational studies in Epidemiology (STROBE) statement: Guidelines for reporting observational studies.. PLoS Med..

[7] Asawa N, Bulbule N, Kakade D, Shah R (2015). Angulated implants: an alternative to bone augmentation and sinus lift procedure: systematic review.. Journal of clinical and diagnostic research..

[8] Egbert N, Ahuja S, Selecman A, Wicks R (2017). Angulated Implants for Fabrication of Implant Supported Fixed Partial Denture in the Maxilla.. Journal of dentistry (Shiraz, Iran)..

[9] Tulbneruo Tan WL, Wong TLT, Wong MCM, Lang NP (2012). A systematic review of postextractional alveolar hard and soft tissue dimensional changes in humans.. Clin Oral Implants Res..

[10] Van der Weijden F, Dell'Acqua F, Slot DE (2009). Alveolar bone dimensional changes of post-extraction sockets in humans: a systematic review.. J Clin Periodontol..

[11] Nielsen HB, Starch-Jensen T (2021). Lateral ridge augmentation in the posterior part of the mandible with an autogenous bone block graft harvested from the ascending mandibular ramus. A 10-year retrospective study.. J Stomatol Oral Maxillofac Surg..

[12] Von Arx T, Buser D (2006). Horizontal ridge augmentation using autogenous block grafts and the guided bone regeneration technique with collagen membranes: a clinical study with 42 patients.. Clin Oral Implants Res..

[13] Mordenfeld A, Aludden H, Starch-Jensen T (2017). Lateral ridge augmentation with two different ratios of deproteinized bovine bone and autogenous bone: a 2- year follow-up of a randomized and controlled trial.. Clin Implant Dent Relat Res..

[14] Barbu HM, Iancu SA, Rapani A, Stacchi C (2021). Guided Bone Regeneration with Concentrated Growth Factor Enriched Bone Graft Matrix (Sticky Bone) *vs*. Bone-Shell Technique in Horizontal Ridge Augmentation: A Retrospective Study.. J Clin Med..

[15] Hartlev J, Nørholt S E, Schou S, Isidor F (2021). Pain after mandibular ramus block harvesting and lateral ridge augmentation with and without involvement of platelet-rich fibrin: a randomized controlled trial.. International Journal of Oral and Maxillofacial Surgery..

[16] Cordaro L, Torsello F, Miuccio MT, Mirisola di Torresanto V, Eliopoulos D (2011). Mandibular bone harvesting for alveolar reconstruction and implant placement: subjective and objective cross-sectional evaluation of donor and recipient site up to 4 years.. Clin. Oral Impl. Res..

[17] Atieh MA, Alsabeeha NH, Tawse-Smith A, Faggion CM, Duncan WJ (2015). Piezoelectric surgery *vs*. rotary instruments for lateral maxillary sinus floor elevation: a systematic review and meta-analysis of intra- and postoperative complications.. Int J Oral Maxillofac Implants..

[18] Stacchi C, Vercellotti T, Toschetti A, Speroni S (2015). Intraoperative complications during sinus floor elevation using two different ultrasonic approaches: a two-center, randomized, controlled clinical trial.. Clin Implant Dent Relat Res..

[19] Magrin GL, Sigua-Rodriguez EA, Goulart DR, Asprino L (2015). Piezosurgery in Bone Augmentation Procedures Previous to Dental Implant Surgery: A Review of the Literature.. Open Dent J..

[20] Sakkas A, Schramm A, Winter K, Wilde F (2018). Risk factors for postoperative complications after procedures for autologous bone augmentation from different donor sites.. Journal of Cranio-Maxillofacial Surgery..

[21] Pikos MA (2005). Mandibular Block Autografts for Alveolar Ridge Augmentation.. Atlas of the Oral and Maxillofacial Surgery Clinics..

[22] Carlsen A, Gorst-Rasmussen A, Jensen T (2013). Donor Site Morbidity Associated With Autogenous Bone Harvesting From the Ascending Mandibular Ramus.. Implant Dentistry..

[23] Carl E, Misch CE, Resnik R (2017). Misch's Avoiding Complications in Oral Implantology.

[24] Sezavar M, Mesgarzadeh V, Shafayifard S, Soleimanpour MR (2015). Management of Bone Grafting Complications in Advanced Implant Surgery.. A Textbook of Advanced Oral and Maxillofacial Surgery Volume..

[25] Titsinides S, Agrogiannis G, Karatzas T (2019). Bone grafting materials in dentoalveolar reconstruction: A comprehensive review.. The Japanese dental science review..

